# The transcription regulator ChpA affects the global transcriptome including quorum sensing‐dependent genes in *Ralstonia pseudosolanacearum* strain OE1‐1

**DOI:** 10.1111/mpp.13374

**Published:** 2023-07-14

**Authors:** Chika Takemura, Wakana Senuma, Masayuki Tsuzuki, Yuki Terazawa, Kanako Inoue, Masanao Sato, Akinori Kiba, Kouhei Ohnishi, Kenji Kai, Yasufumi Hikichi

**Affiliations:** ^1^ Faculty of Agriculture and Marine Science Kochi University Nankoku Japan; ^2^ Research Center for Ultra‐High Voltage Electron Microscopy Osaka University Ibaraki Japan; ^3^ Graduate School of Agriculture Hokkaido University Sapporo Japan; ^4^ Graduate School of Agriculture Osaka Metropolitan University Sakai Japan; ^5^ Present address: Kochi Prefectural Agriculture Research Center Nankoku Japan; ^6^ Present address: Central Research Institute, Ishihara Sangyo Kaisha, Ltd. Kusatsu Japan; ^7^ Present address: Kumamoto Experimental Station, Sumika Agrotech Co., Ltd. Kikuchi Japan; ^8^ Present address: Division of Biological Sciences Plant Immunity, Nara Institute of Science and Technology Ikoma Japan

**Keywords:** ChpA, quorum sensing, *Ralstonia pseudosolanacearum*, virulence

## Abstract

The gram‐negative plant‐pathogenic β‐proteobacterium *Ralstonia pseudosolanacearum* strain OE1‐1 produces methyl 3‐hydroxymyristate as a quorum sensing (QS) signal through methyltransferase PhcB and senses the chemical via the sensor histidine kinase PhcS. This leads to activation of the LysR family transcription regulator PhcA, which regulates the genes (QS‐dependent genes) responsible for QS‐dependent phenotypes, including virulence. The transcription regulator ChpA, which possesses a response regulator receiver domain and also a hybrid sensor histidine kinase/response regulator phosphore‐acceptor domain but lacks a DNA‐binding domain, is reportedly involved in QS‐dependent biofilm formation and virulence of *R. pseudosolanacearum* strain GMI1000. To explore the function of ChpA in QS of OE1‐1, we generated a *chpA*‐deletion mutant (Δ*chpA*) and revealed that the *chpA* deletion leads to significantly altered QS‐dependent phenotypes. Furthermore, Δ*chpA* exhibited a loss in its infectivity in xylem vessels of tomato plant roots, losing virulence on tomato plants, similar to the *phcA*‐deletion mutant (Δ*phcA*). Transcriptome analysis showed that the transcript levels of *phcB*, *phcQ*, *phcR*, and *phcA* in Δ*chpA* were comparable to those in OE1‐1. However, the transcript levels of 89.9% and 88.9% of positively and negatively QS‐dependent genes, respectively, were significantly altered in Δ*chpA* compared with OE1‐1. Furthermore, the transcript levels of these genes in Δ*chpA* were positively correlated with those in Δ*phcA*. Together, our results suggest that ChpA is involved in the regulation of these QS‐dependent genes, thereby contributing to the behaviour in host plant roots and virulence of OE1‐1.

## Graphical abstract

The transcription regulator ChpA is involved in the regulation of quorum sensing‐dependent genes of *Ralstonia pseudosolanacearum* strain OE1‐1, thereby contributing to its behaviour in host plant roots and virulence.

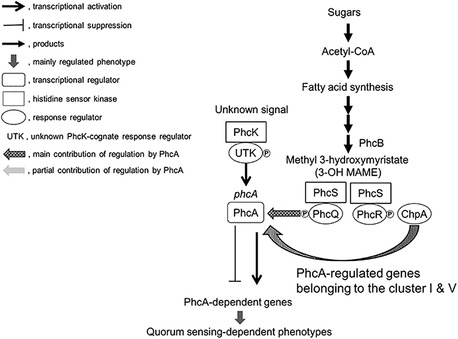

## 1 INTRODUCTION

Bacteria monitor quorum sensing (QS) signals to track changes in abundance and to activate QS for the synchronous control of the expression of genes beneficial for vigorous replication, adaptation to environmental conditions, and virulence (Ham, 2013; Rutherford & Bassler, 2012). The soilborne gram‐negative β‐proteobacterium *Ralstonia solanacearum* species complex (RSSC; Remenant et al., 2010) infects more than 250 plant species in over 50 families, causing a devastating bacterial wilt disease that damages crop production worldwide (Mansfield et al., 2012). A phylotype I strain of RSSC, *R. pseudosolanacearum* OE1‐1 (Kanda et al., 2003), first attaches to the surface of the epidermis in meristematic zones and elongation zones in roots of 4‐day‐old tomato seedlings without secondary roots and then colonizes intercellular spaces between the epidermis and cortex, activating QS (Inoue et al., 2023). This leads to degradation of the cell wall of cortical cells adjacent to the epidermis by plant cell wall‐degrading enzymes such as cellobiohydrolase (Liu et al., 2005), endoglucanase (Roberts et al., 1988), and pectin methylesterase (Tans‐Kersten et al., 1998), which are produced in the active state of QS and secreted through type II secretion machinery (Tsujimoto et al., 2008), and infection of the cell wall‐denatured cortical cells (Inoue et al., 2023). The infection of cell wall‐denatured cortical cells by OE1‐1 leads to further infection of OE1‐1 in xylem vessels. Therefore, QS is required for OE1‐1 virulence.

OE1‐1 produces methyl 3‐hydroxymyristate (3‐OH MAME) as the QS signal (Kai et al., 2015; Ujita et al., 2019). The QS signal is synthesized by the methyltransferase PhcB and is sensed through the sensor histidine kinase PhcS. The 3‐OH MAME sensing through PhcS leads to the phosphorylation of the regulators PhcQ and PhcR; PhcQ and PhcR strongly and partially contribute, respectively, to the regulation of QS‐dependent genes by the LysR family transcription regulator PhcA, which has a conserved structure with an N‐terminal DNA‐binding helix‐turn‐helix motif and a C‐terminal co‐inducer‐binding domain (Maddocks & Oyston, 2008; Takemura et al., 2021). The sensor histidine kinase PhcK is required for full expression of *phcA*, independent of 3‐OH MAME sensing (Senuma et al., 2020). In the active state of QS, PhcA regulates QS‐dependent genes responsible for QS‐dependent phenotypes including virulence and induces the production of the virulence‐related aryl‐furanone secondary metabolites, ralfuranones (Kai et al., 2014; Kai et al., 2016), and the major exopolysaccharide EPS I (Garg et al., 2000; Schell, 2000). These secondary metabolites are associated with the feedback loop of QS‐dependent gene regulation (Hayashi, Senuma, et al., 2019; Mori et al., 2018). In the QS active state, expression of *lecM*, encoding the lectin LecM, is induced, which affects the activation of QS through regulating the stability of extracellularly secreted 3‐OH MAME (Hayashi, Kai, et al., 2019). Furthermore, QS‐inducible β‐1,4‐cellobiohydrolase is involved not only in plant cell wall degradation (Liu et al., 2005; Tsujimoto et al., 2008), but also in the full expression of *phcA*, thereby contributing to the QS feedback loop and virulence of OE1‐1 (Senuma et al., 2023).

The transcription regulator ChpA reportedly regulates l‐glutamic acid signalling‐related genes in a phylotype I strain of RSSC, *R. pseudosolanacearum* GMI1000 (Shen et al., 2020), which produces 3‐OH MAME as the QS signal (Kai et al., 2015). The deduced amino acid sequence of ChpA based on its nucleotide sequence confirms the presence of not only a response regulator receiver domain but also a hybrid sensor histidine kinase/response regulator phosphor‐acceptor receiver domain. ChpA is conserved among RSSC strains. ChpA is an intracellular soluble protein that lacks transmembrane helices and a DNA‐binding domain (Corral et al., 2020; Shen et al., 2020). ChpA is involved in the QS‐dependent regulation of biofilm formation (Corral et al., 2020), chemotaxis (Corral et al., 2020), exopolysaccharide production (Shen et al., 2020), cellulase activity (Shen et al., 2020), and swarming activity (Corral et al., 2020), but not the swimming activity (Corral et al., 2020) of GMI1000. Furthermore, deletion of *chpA* in GMI1000 also leads to significantly reduced virulence (Corral et al., 2020; Shen et al., 2020). However, how ChpA regulates these QS‐dependent phenotypes of RSSC including virulence remains unclear.

To elucidate the virulence mechanisms of OE1‐1, it is important to comprehensively analyse its QS signalling pathway. Here, to elucidate the role of ChpA in QS‐dependent phenotypes including their virulence on tomato plants, we analysed the transcriptomes of a *chpA*‐deletion mutant (Δ*chpA*, Table 1) from OE1‐1 and a transformant of Δ*chpA* with native *chpA* (*chpA*‐comp, Table 1) using reverse transcription‐quantitative real‐time PCR (RT‐qPCR) and RNA‐sequencing (RNA‐seq).

**TABLE 1:** Strains and plasmids used in this study.

	Relevant characteristics	Source
Plasmids		
pUC118	Amp^r^	Takara Bio
pK18mobsacB	Km^r^, *oriT* (RP4), *sacB*, *lacZα*	Kvitko and Collmer (2011)
pUC18‐mini‐Tn*7*T‐Gm	Gm^r^	Choi et al. (2005)
pTNS2	Helper plasmid carrying the Tn*7* transposase gene	Choi et al. (2005)
pdelta‐chpA	pK18mobsacB derivative carrying a 1.4‐kbp DNA fragment for *chpA* deletion, Km^r^	This study
pUC18‐mini‐Tn*7*T‐Gm‐chpA	pUC18‐mini‐Tn*7*T‐Gm derivative carrying a 7.5‐kbp fragment for *chpA* complementation, Gm^r^	This study
*Escherichia coli* strain		
DH5α	*recA1 endA1 gyrA96 thi‐1 hsdR17 supE44* Δ(*lac*)*U169*(ϕ*80lac*ΔM15)	Takara Bio
*Ralstonia solanacearum* strains		
OE1‐1	Wild‐type strain, phylotype I, race 1, biovar 4	Kanda et al. (2003)
Δ*phcB*	*phcB*‐deletion mutant of OE1‐1	Kai et al. (2015)
Δ*phcA*	*phcA*‐deletion mutant of OE1‐1	Mori et al. (2016)
Δ*phcK*	*phcK*‐deletion mutant of OE1‐1	Senuma et al. (2020)
Δ*phcR*	*phcR*‐deletion mutant of OE1‐1	Takemura et al. (2021)
Δ*phcQ*	*phcQ*‐deletion mutant of OE1‐1	Takemura et al. (2021)
Δ*chpA*	*chpA*‐deletion mutant of OE1‐1	This study
*chpA*‐comp	A transformant of Δ*chpA* with pUC18‐mini‐Tn*7*T‐Gm‐chpA containing native *chpA*, Gm^r^	This study

## 2 RESULTS

### 2.1 Deletion of *chpA* significantly altered QS‐dependent phenotypes

**FIGURE 1:**
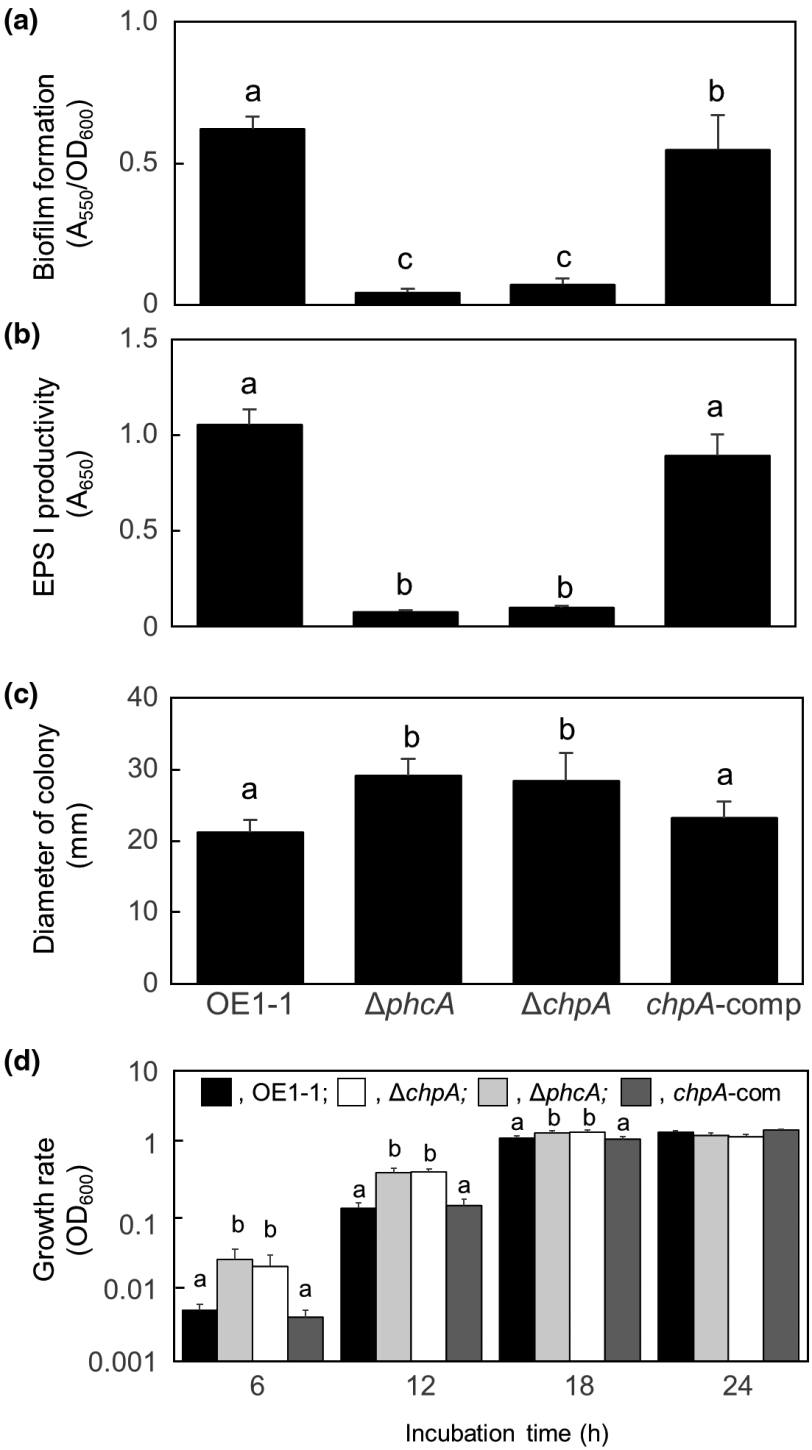
Biofilm formation (a), production of the major exopolysaccharide EPS I (b), swimming motility (c), and the in vitro growth rate (d) of *Ralstonia pseudosolanacearum* strain OE1‐1, the *phcA*‐deletion (Δ*phcA*) and *chpA*‐deletion (Δc*hpA*) mutants, and Δ*chpA* transformed with native *chpA* (*chpA*‐comp). (a) Biofilm formation of *R. pseudosolanacearum* incubated in quarter‐strength M63 medium was quantified by measuring absorbance at 550 nm (A_550_) and normalized to the number of cells (optical density at 600 nm, OD_600_). Three replicate experiments were conducted using independent samples, with seven technical replicates per experiment. (b) *R. pseudosolanacearum* strains were incubated on quarter‐strength M63 medium solidified with 0.25% wt/vol agar. EPS I content of the supernatant was quantified by ELISA. Three replicate experiments were conducted using independent samples, with seven technical replicates per experiment. (c) *R. pseudosolanacearum* strains were grown on quarter‐strength M63 medium solidified with 0.25% wt/vol agar. Three replicate experiments were conducted using independent samples, with five technical replicates per experiment. (d) Overnight cultures of *R. pseudosolanacearum* strains were diluted to OD_600_ = 0.01 with quarter‐strength M63 and grown with shaking. The in vitro growth rate of bacterial strains was quantified based on OD_600_ of the bacterial culture. Three replicate experiments were conducted using independent samples, with three technical replicates per experiment. Bars indicate standard error. Means were analysed for significant differences between *R. pseudosolanacearum* strains by analysis of variance followed by Tukey–Kramer's honestly significant difference test. Statistically significant differences are indicated by different lowercase letters (*p* < 0.05).

We created Δ*chpA* using strain OE1‐1 and analysed its QS‐dependent phenotypes (Figure S1). To determine the effect of ChpA on QS, we first examined the in vitro biofilm formation of *R. pseudosolanacearum* strains grown without shaking in quarter‐strength M63. Compared with OE1‐1, Δ*chpA* produced significantly less biofilm, similar to the *phcA*‐deletion mutant (Δ*phcA*; Table 1; Mori et al., 2016) (*p* < 0.05, *t* test; Figure 1a). Compared with OE1‐1, Δ*chpA* produced significantly less EPS I (*p* < 0.05, *t* test; Figure 1b), like Δ*phcA*. Similar to Δ*phcA*, Δ*chpA* exhibited significantly enhanced swimming motility compared with OE1‐1 (*p* < 0.05, *t* test; Figure 1c).

Next, we transformed Δ*chpA* with pUC18‐mini‐Tn*7*T‐Gm‐chpA (Table 1) harbouring the native *chpA* to generate a complemented Δ*chpA* strain (*chpA*‐comp; Table 1). The transformation of Δ*chpA* with *chpA* restored biofilm formation (Figure 1a), EPS I production (Figure 1b), and swimming motility (Figure 1c). These results indicate that ChpA is involved in the regulation of QS‐dependent phenotypes.

It has been reported that the *phcA* mutant exhibits faster in vitro growth than the parent strain (Khokhani et al., 2017; Peyraud et al., 2016), suggesting that the in vitro growth rate of RSSC is negatively associated with QS activation. Compared with OE1‐1 and *chpA*‐comp, Δ*chpA* exhibited faster in vitro growth, like Δ*phcA* (*p* < 0.05, *t* test; Figure 1d).

### 2.2 Deletion of *chpA* significantly affected the expression levels of QS‐dependent genes

**FIGURE 2:**
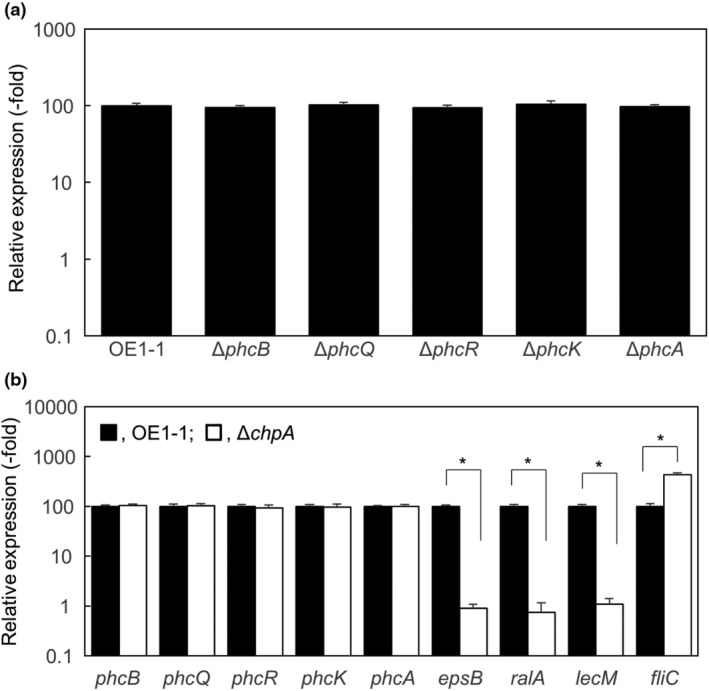
Gene transcript levels in wild‐type and mutant strains. (a) Transcript levels of *chpA* in *Ralstonia pseudosolanacearum* strain OE1‐1 and the *phcB*‐deletion (Δ*phcB*), *phcQ*‐deletion (Δ*phcQ*), *phcR*‐deletion (Δ*phcR*), *phcK*‐deletion (Δ*phcK*), and *phcA*‐deletion (Δ*phcA*) mutants. (b) Transcript levels of quorum sensing (QS)‐related genes *phcB*, *phcQ*, *phcR*, *phcK*, and *phcA*, and QS‐dependent genes *epsB*, *ralA*, *lecM*, and *fliC*, in OE1‐1 and the *chpA*‐deletion mutant (Δ*chpA*). Gene transcript levels were normalized to that of *rpoD*. The experiments were performed with three biological replicates and two technical replicates. Bars indicate standard errors. Asterisks indicate a significant difference from OE1‐1 (*p* < 0.05, *t* test).

The expression of three genes—*epsB*, a component of the *eps* operon that is required for EPS I biosynthesis (Huang & Schell, 1995); *ralA*, encoding ralfuranone synthase (Kai et al., 2014); and *lecM* (Mori et al., 2016)—is dependent on QS. The expression of the flagellar motility‐related gene *fliC*, encoding flagellin, is suppressed during QS (Tans‐Kersten et al., 2001). Using RT‐qPCR, we analysed the transcript levels of these genes in the *R. pseudosolanacearum* strains grown in quarter‐strength M63 medium until OD_600_ = 0.3. The transcript levels of *epsB*, *ralA*, and *lecM* were significantly lower in Δ*chpA* than in OE1‐1 (*p* < 0.05, *t* test; Figure 2a), while that of *fliC* was higher in Δ*chpA* than in OE1‐1 (*p* < 0.05, *t* test; Figure 2a).

### 2.3 Deletion of *chpA* did not influence the regulation of QS‐related genes

The QS‐dependent phenotypes differed significantly between Δ*chpA* and OE1‐1. To explore the reason for these differences, we conducted RT‐qPCR analyses to determine the transcript levels of QS‐related genes, *phcB*, *phcQ*, *phcR*, *phcK*, and *phcA*, in *R. pseudosolanacearum* strains grown in quarter‐strength M63 medium until OD_600_ = 0.3. The transcript levels of these QS‐related genes did not differ significantly between Δ*chpA* and OE1‐1 (Figure 2a). Furthermore, the *chpA* transcript levels were not significantly different among OE1‐1, the *phcB*‐deletion mutant (Δ*phcB*; Table 1; Kai et al., 2015), the *phcQ*‐deletion mutant (Δ*phcQ*; Table 1; Takemura et al., 2021), the *phcR*‐deletion mutant (Δ*phcR*; Table 1; Takemura et al., 2021), the *phcK*‐deletion mutant (Δ*phcK*; Table 1; Senuma et al., 2020), and Δ*phcA* (Figure 2b).

### 2.4 Deletion of *chpA* resulted in a loss of virulence

**FIGURE 3:**
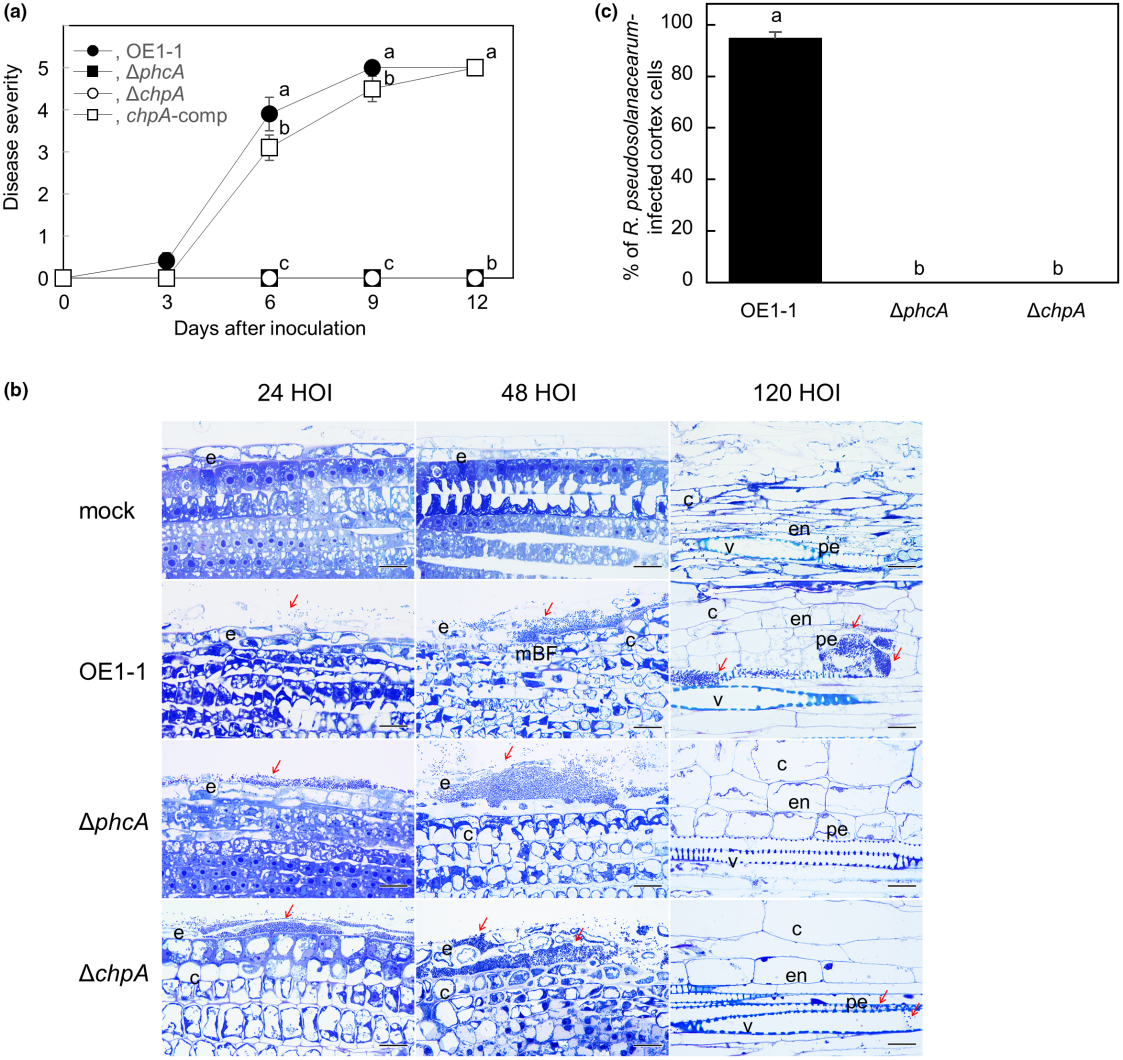
Virulence of *Ralstonia pseudosolanacearum* strains on tomato plants (a), microscopic observation of roots of 4‐day‐old tomato seedlings co‐incubated with *R. pseudosolanacearum* strains (b), and percentages of cell wall‐degraded cortex cells infected with *R. pseudosolanacearum* strains in the meristematic zone in roots of 4‐day‐old tomato seedlings after 48 h of co‐incubation (HOI) with *R. pseudosolanacearum* strains (c). (a) Eight‐week‐old tomato plants were inoculated with *R. pseudosolanacearum* strain OE1‐1, the *phcA*‐deletion (Δ*phcA*) and *chpA*‐deletion (Δ*chpA*) mutants, and Δ*chpA* transformed with native *chpA* (*chpA*‐comp). Plants were rated according to the following disease index scale: 0, no wilting; 1, 1%–25% wilting; 2, 26%–50% wilting; 3, 51%–75% wilting; 4, 76%–99% wilting; 5, dead. For each bacterial strain, three independent groups were tested, with 12 technical replicates per group. Bars indicate standard error. Means were analysed for significant differences between *R. pseudosolanacearum* strains by analysis of variance followed by Tukey–Kramer's honestly significant difference test. Statistically significant differences are indicated by different lowercase letters (*p* < 0.05). (b) Mock and 24, 48, and 120 HOI with *R. pseudosolanacearum* strains OE1‐1, Δ*phcA*, and Δ*chpA*. Longitudinal semithin resin sections were stained with toluidine blue. Red arrows indicate bacterial cells. Bars indicate 20 μm. e, epidermal cell; c, cortical cell; en, endodermis cell; pe, pericycle cell; v, xylem vessel; mBF, mushroom‐shaped biofilm. (c) Micrographs of toluidine blue‐dyed serial semi‐thin sections (800 nm thick) from the meristematic zone in roots were observed. The experiment was repeated five times, each with 100 technical replicates. Bars indicate standard error. Means were analysed for significant differences between *R. pseudosolanacearum* strains by analysis of variance followed by Tukey–Kramer's honestly significant difference test. Statistically significant differences are indicated by different lowercase letters (*p* < 0.05).

To investigate the effects of *chpA* on the virulence of OE1‐1, we inoculated 8‐week‐old tomato plants with *R. pseudosolanacearum* strains by the root‐dip method. The plants inoculated with OE1‐1 exhibited wilt symptoms at 5 days after inoculation (DAI) and died by 9 DAI (Figure 3a). Like Δ*phcA*, Δ*chpA* was not virulent on tomato plants.

To investigate the behaviour of Δ*chpA* in roots of 4‐day‐old tomato seedlings using an in vitro pathosystem (Inoue et al., 2023), we observed toluidine blue‐dyed sections of tomato roots co‐incubated with Δ*chpA* under an optical microscope. After 24 h of co‐incubation (HOI), we observed detached epidermis in meristematic zones and elongation zones of tomato roots co‐incubated with Δ*chpA*, similar to those co‐incubated with OE1‐1 or with Δ*phcA* (Figure 3b). After 48 HOI, cell walls of cortical cells adjacent to the epidermis of tomato roots co‐incubated with OE1‐1 but not Δ*chpA* or Δ*phcA* were degraded. Furthermore, no Δ*chpA* or Δ*phcA* cells were present in cortical cells (Figure 3c). After 120 HOI, no Δ*phcA* cells were observed in xylem vessels. However, after 120 HOI a smaller number of Δ*chpA* cells was observed in xylem vessels compared to the number of OE1‐1 cells (Figure 3b).

### 2.5 RNA‐seq transcriptome analysis of *R. pseudosolanacearum* strains

To comprehensively analyse the effects of *chpA* deletion on the regulation of QS‐dependent genes, we performed RNA‐seq transcriptome analyses of *R. pseudosolanacearum* strains grown in quarter‐strength M63 medium until OD_600_ = 0.3. Mapping of RNA‐seq reads of OE1‐1 to the GMI1000 genome (Salanoubat et al., 2002) resulted in the identification of 4311 protein‐coding transcripts (Table S1). The following thresholds were applied to identify genes with significant changes in transcript levels: *q* < 0.05 and log_2_(fold change) ≥ |2|. To identify positively and negatively QS‐dependent genes, we compared their transcript levels in OE1‐1 with those in Δ*phcB* and Δ*phcA*. In both Δ*phcB* and Δ*phcA*, 315 and 180 genes commonly exhibited significant down‐regulation and up‐regulation, respectively, and were thus inferred to be positively and negatively QS‐dependent genes, respectively (Table S2).

**FIGURE 4:**
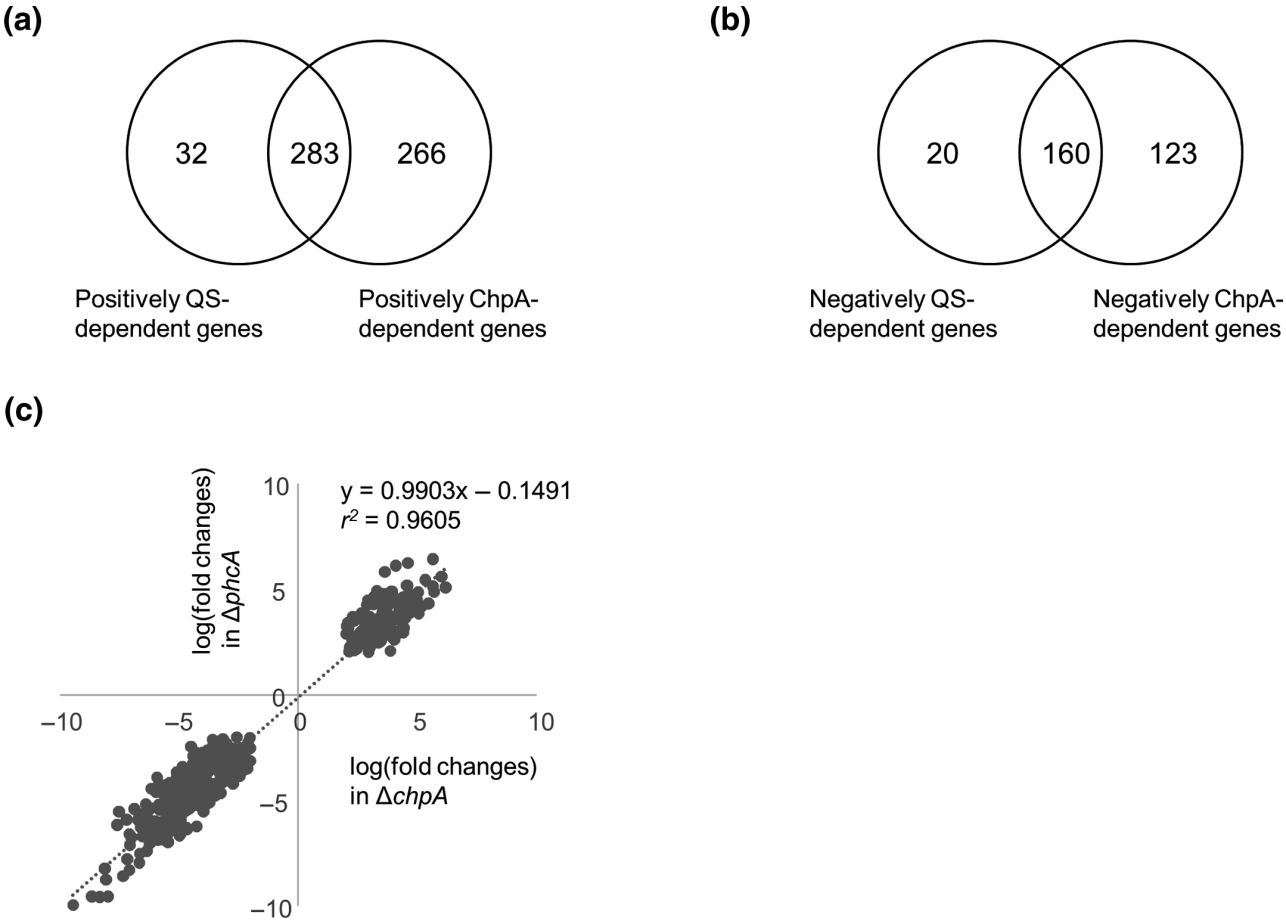
RNA‐sequencing transcriptome analysis of *Ralstonia pseudosolanacearum* strains grown in quarter‐strength M63 medium until OD_600_ = 0.3. (a) Numbers of genes with transcript‐level log_2_(fold change) ≤ −2 in the *chpA*‐deletion (Δ*chpA*) and *phcA*‐deletion (Δ*phcA*) mutants relative to their transcript levels in OE1‐1 (*q* < 0.05). (b) Number of genes with transcript‐level log_2_(fold change) ≥ 2 in Δ*chpA* and Δ*phcA* relative to their transcript levels in OE1‐1 (*q* < 0.05). (c) Correlation of transcript levels of ChpA‐dependent genes among quorum sensing (QS)‐dependent genes between *R. pseudosolanacearum* mutants: Δ*chpA* versus Δ*phcA*.

The transcriptome analyses revealed that 549 genes (positively ChpA‐dependent genes) exhibited significant down‐regulation in Δ*chpA* compared with their transcript levels in OE1‐1, while 333 genes (negatively ChpA‐dependent genes) were significantly up‐regulated in Δ*chpA* (Table S3). Among the positively ChpA‐dependent genes, 283 were positively QS‐dependent genes (Figure 4a, Table S3). Among the negatively ChpA‐dependent genes, 160 were negatively QS‐dependent genes (Figure 4b, Table S3). The transcript levels of ChpA‐dependent genes among QS‐dependent genes were strongly and positively correlated between Δ*chpA* and Δ*phcA* (*y* = 0.9903*x* − 0.1491, *r* ^2^ = 0.9605; where *y* is log[fold change in Δ*phcA*] and *x* is log[fold change in Δ*chpA*]; Figure 4c). These results indicate that ChpA is strongly involved in the regulation of 283 genes (89.9%) and 160 genes (88.9%) among positively and negatively QS‐dependent genes, respectively.

### 2.6 ChpA affected the genome‐wide transcriptome for some gene sets in a PhcA‐independent manner

**FIGURE 5:**
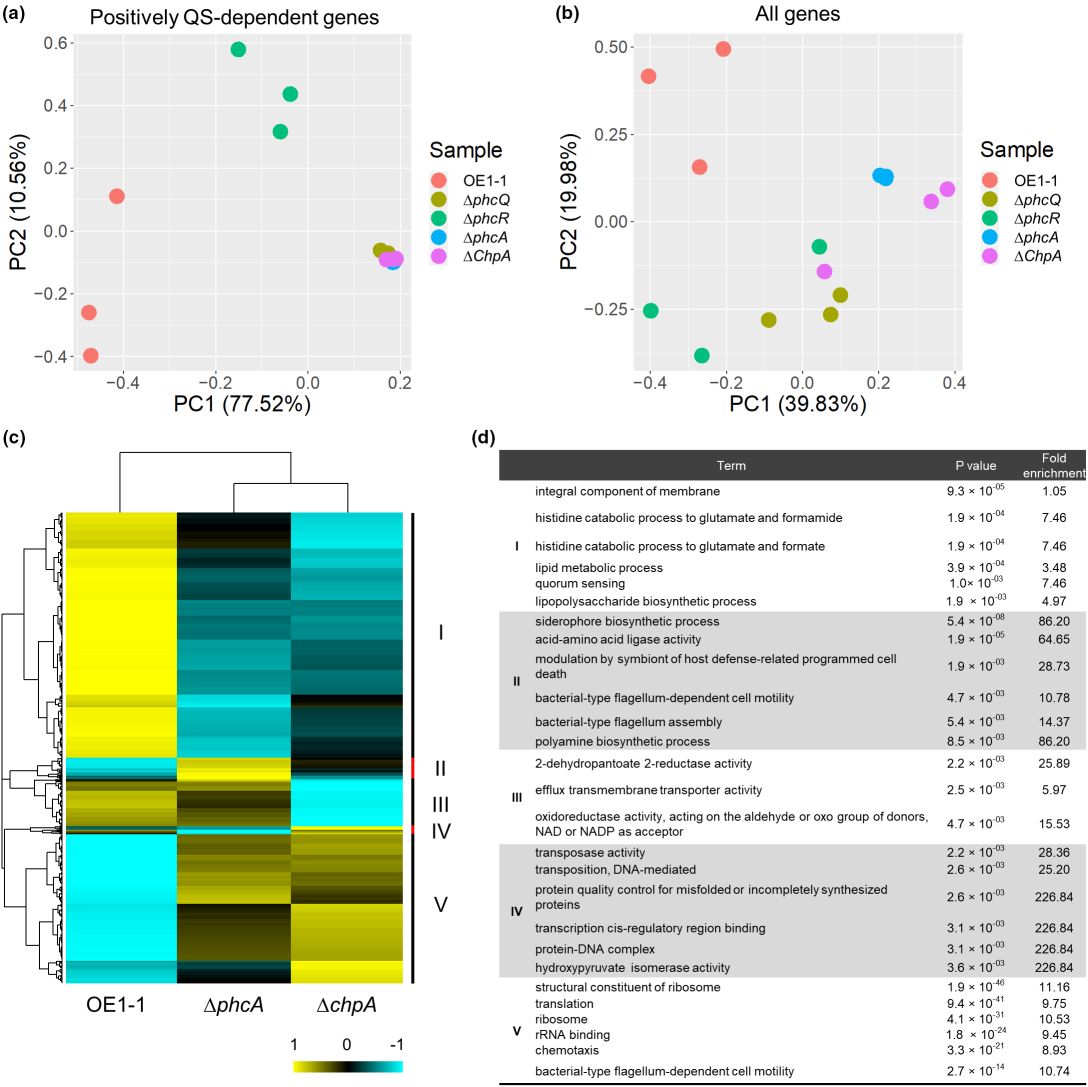
Global effect of ChpA on gene expression in *Ralstonia pseudosolanacearum*. A principal component analysis plot for the transcriptome data of positively quorum sensing (QS)‐dependent genes (a) and all genes (b) in OE1‐1 and *phcA*‐deletion (Δ*phcA*), *phcR*‐deletion (Δ*phcR*), *phcQ*‐deletion (Δ*phcQ*), and *chpA*‐deletion (Δ*chpA*) mutants. (c) Hierarchical clustering and heatmap analysis of relative transcript levels of genes that were differentially expressed between at least two genotypes (OE1‐1, Δ*phcA*, and Δ*chpA*) based on RNA‐sequencing data with the samples grown in quarter‐strength M63 medium until OD_600_ = 0.3. Values are averages of three replicates per strain. Hierarchical clustering by the complete linkage method was applied to *z*‐scores calculated with normalized log_2_(fold change) values of all normalized mean expression values (counts per million) using the hclust R package. The heatmap was created with the R package pheatmap. (d) GO terms enriched in each cluster shown in (c).

To determine the global effect of ChpA on the *R. pseudosolanacearum* transcriptome, we further performed principal component analysis (PCA) on a certain gene set with the RNA‐seq data of Δ*chpA* as well as Δ*phcA*. The resultant loadings for positively QS‐dependent genes indicated that ChpA contributes to positively PhcA‐regulated gene expression in a similar manner to PhcA (Figure 5a). On the other hand, a PCA plot for all genes or negatively QS‐dependent genes suggested that the effect of ChpA on the transcriptome is different from that of PhcA, suggesting the effect of ChpA on transcriptional control is distinct for other gene sets (Figures 5b and S2). Hierarchical clustering with a heatmap representing the global gene expression pattern in OE1‐1, ∆*phcA*, and ∆*chpA* showed that the effect of ChpA on the gene expression pattern is close to that of PhcA, though they are different for some gene sets (Figure 5c). The Gene Ontology (GO) terms enriched in cluster I include terms related to QS‐related genes like “quorum sensing (GO:0009372)” and “lipopolysaccharide biosynthesis process (GO:0009103)”, with a similar expression pattern between ∆*phcA* and ∆*chpA* (Figure 5c,d). Also, the cluster V genes, which are enriched in negatively QS‐dependent genes, showed decreased expression in ∆*phcA* and ∆*chpA*, including terms like “chemotaxis (GO:0006935)” and “bacterial‐type flagellum‐dependent cell motility (GO:0071973)”. Interestingly, some gene sets belonging to cluster II, III, or IV showed either ∆*phcA‐* or ∆*chpA*‐specific gene expression (Figure 5c). Cluster II, with genes up‐regulated only in ∆*phcA*, showed significant enrichment of the terms “siderophore (GO:0019290)” and “acid‐amino acid ligase activity (GO:0016881)", whereas cluster III showed ∆*chpA*‐specific down‐regulation including the enriched terms “2‐dehydropantoate 2‐reductase activity (GO:0008677)” and “efflux transmembrane transporter activity (GO:0015562)” (Figure 5c,d). These results suggest that ChpA has an effect on transcriptional control in a similar manner to PhcA on QS‐dependent genes, though it also has PhcA‐independent effects on the *R. pseudosolanacearum* transcriptome and vice versa for PhcA.

### 2.7 ChpA is involved in the positive control of siderophore‐mediated iron acquisition in a QS‐independent manner

**FIGURE 6:**
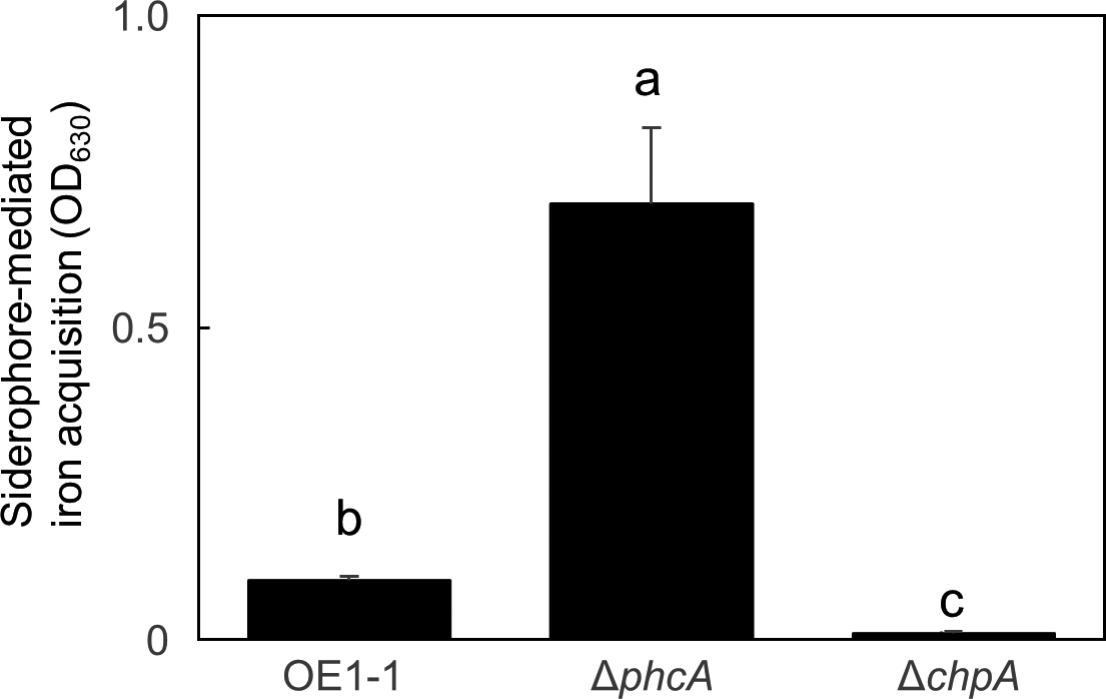
Siderophore‐mediated iron acquisition activity of *Ralstonia pseudosolanacearum* strain OE1‐1 and the *phcA*‐deletion (Δ*phcA*) and *chpA*‐deletion (Δ*chpA*) mutants grown in PY medium. Three replicate experiments were conducted using independent samples, with eight technical replicates per assay. Means were analysed for significant differences between *R. pseudosolanacearum* strains by analysis of variance followed by Tukey–Kramer's honestly significant difference test. Statistically significant differences are indicated by different lowercase letters (*p* < 0.05).

RSSC strains produce micacocidin (Kreutzer et al., 2011) and staphyloferrin B (Bhatt & Denny, 2004) as siderophores to acquire iron from the extracellular environment. PhcA negatively controls siderophore‐mediated iron acquisition (Takemura et al., 2021). The GO terms enriched in cluster II showed ∆*phcA‐* but not ∆*chpA*‐specific gene expression (Figure 5c), and cluster II, with genes up‐regulated only in ∆*phcA*, showed significant enrichment of siderophore‐related terms (Figure 5d). To determine the effect of ChpA on siderophore‐mediated iron acquisition, we measured iron acquisition activity in different *R. pseudosolanacearum* strains. Compared with OE1‐1, Δ*phcA* showed even higher siderophore‐mediated iron acquisition activity (*p* < 0.05, *t* test; Figure 6). In contrast, Δ*chpA* had significantly lower siderophore‐mediated iron acquisition activity than OE1‐1.

### 2.8 ChpA contributes to the regulation of genes involved in twitching motility governed by type IV pili in a QS‐independent manner

**FIGURE 7:**
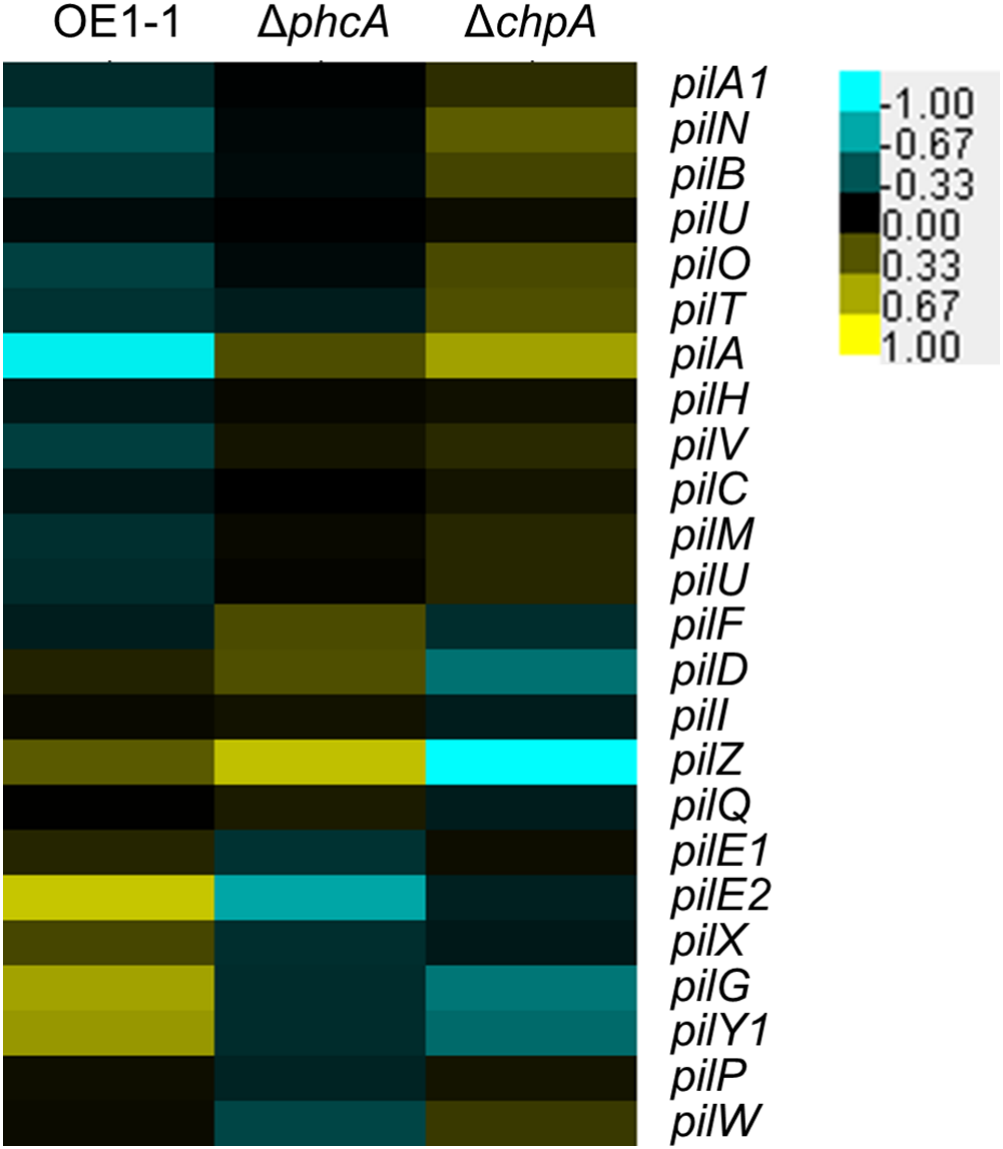
Hierarchical clustering of relative transcript levels of genes involved in twitching motility governed by type IV pilus genes in *Ralstonia pseudosolanacearum* strain OE1‐1 and the *phcA*‐deletion (Δ*phcA*) and *chpA*‐deletion (Δ*chpA*) mutants grown in quarter‐strength M63 medium until OD_600_ = 0.3. Based on RNA‐sequencing transcriptome data for *R. pseudosolanacearum* strains, fragments per kilobase of open reading frame per million fragments mapped values from *R. pseudosolanacearum* strains OE1‐1, Δ*phcA*, and Δ*chpA* were normalized prior to analysis of differentially expressed genes. Hierarchical clustering of all normalized mean expression values (counts per million) was performed using Cluster v. 3.0 software. Heatmaps were created with TreeView.

Twitching movement is impaired in the *chpA* mutant generated from GMI1000 (Corral et al., 2020). To analyse the influence of *chpA* deletion on the transcript levels of genes involved in twitching motility governed by type IV pili, we carried out hierarchical clustering of Δ*phcA* and Δ*chpA* as well as OE1‐1, based on their relative transcript levels normalized against those of genes involved in twitching motility governed by type IV pili (Table S4). The expression patterns of *pilN*, *pilB*, *pilO*, *pilT*, *pilA*, *pilD*, *pilZ*, *pilK*, and *pilY1* differed between Δ*chpA* and OE1‐1 (Figure 7). Furthermore, the expression patterns of *pilD*, *pilZ*, and *pilW* differed between Δ*chpA* and Δ*phcA*.

### 2.9 Swimming motility of the Δ*chpA* mutant is enhanced compared with that of OE1‐1 when incubated on modified CPG medium with 0.3% agar

**FIGURE 8:**
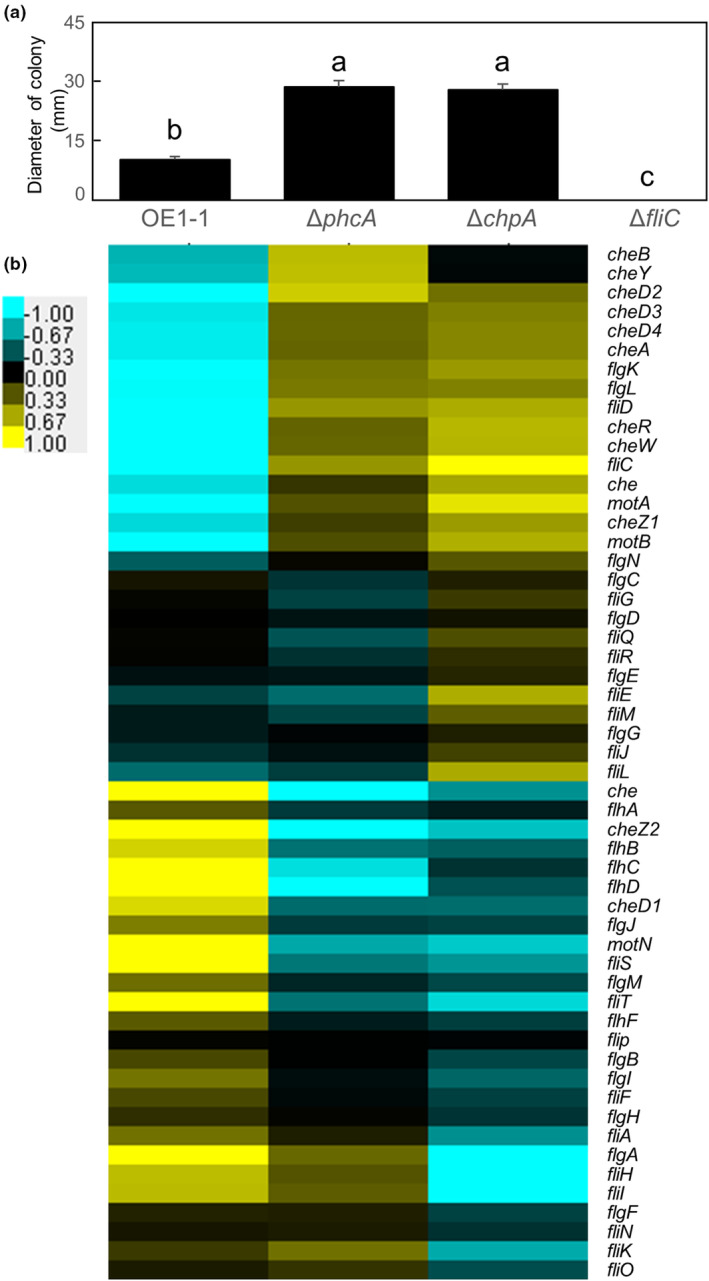
(a) Swimming motility of *Ralstonia pseudosolanacearum* strain OE1‐1 and the *phcA*‐deletion (Δ*phcA*), *chpA*‐deletion (Δ*chpA*), and *fliC*‐deletion (Δ*fliC*) mutants grown on modified CPG medium containing 0.3% wt/vol agar and (b) hierarchical clustering of relative transcript levels of flagellar motility‐related genes in OE1‐1, Δ*phcA*, and Δ*chpA* grown in quarter‐strength M63 medium until OD_600_ = 0.3. (a) The experiment was repeated three times, each with five technical replicates. Bars indicate standard errors. Means were analysed for significant differences between *R. pseudosolanacearum* strains by analysis of variance followed by Tukey–Kramer's honestly significant difference test. Statistically significant differences are indicated by different lowercase letters (*p* < 0.05). (b) Based on RNA‐sequencing transcriptome data for *R. pseudosolanacearum* strains, fragments per kilobase of open reading frame per million fragments mapped values from OE1‐1, Δ*phcA*, and Δ*chpA* were normalized prior to analysis of differentially expressed genes. The average value of three replicates per strain was used. Hierarchical clustering of all normalized mean expression values (counts per million) was performed using Cluster v. 3.0 software. Heatmaps were created with TreeView.

When incubated on modified CPG medium containing 0.3% wt/vol agar, the Δ*chpA* strain generated from GMI1000 exhibits similar swimming motility to that of the parent strain GMI1000 (Corral et al., 2020). In this study, however, the Δ*chpA* strain generated from OE1‐1 exhibited significantly enhanced swimming motility compared with that of OE1‐1 when incubated on quarter‐strength M63 medium containing 0.25% wt/vol agar. We then analysed the swimming motility of Δ*chpA* on modified CPG medium containing 0.3% wt/vol agar. Compared with the swimming motility of OE1‐1, the swimming motility of the *fliC*‐deletion mutant (Δ*fliC*) was significantly reduced, while those of the Δ*chpA* and Δ*phcA* mutants were significantly enhanced (*p* < 0.05, *t* test; Figure 8a).

We carried out hierarchical clustering of Δ*phcA* and Δ*chpA* as well as OE1‐1 cultured on quarter‐strength M63 based on their relative transcript levels normalized against those of swimming motility‐related genes (Table S5). The expression patterns of chemotaxis‐related genes were similar in Δ*chpA* and Δ*phcA*, but those of flagella biogenesis‐related genes differed between Δ*chpA* and Δ*phcA* (Figure 8b).

## 3 DISCUSSION

Recently, we proposed a scenario to explain the QS signalling cascade of OE1‐1 (Takemura et al., 2021). In this scenario, the sensor histidine kinase PhcK is required for full expression of *phcA*, encoding the LysR family transcription regulator PhcA, independently of QS signal production by PhcB (Senuma et al., 2020). PhcQ contributes to the regulation of QS‐dependent genes, in which PhcR is partially involved, dependent on sensing of 3‐OH MAME through the sensor kinase PhcS (Takemura et al., 2021). The regulator ChpA in RSSC strains contains an REC domain and a CheA‐like domain, but it lacks transmembrane helices and a DNA‐binding domain. ChpA is an intracellular soluble protein that is conserved among RSSC strains (Corral et al., 2020; Shen et al., 2020). Results in this study showed that ChpA is involved in the regulation of 89.9% and 88.9% of positively and negatively QS‐dependent genes, respectively, suggesting a large overlap between genes regulated by the global QS regulator PhcA and genes that are regulated by ChpA. It is thus thought that ChpA might participate in the regulation of these genes by PhcA, similar to PhcQ and PhcR, or by alternative transcription regulator(s). The comparative transcriptome analysis showed that some gene sets belonging to cluster II, III, or IV in the hierarchical clustering analysis and the heatmap of relative transcript levels are differentially expressed between at least two genotypes in OE1‐1. Furthermore, Δ*phcA* and Δ*chpA* showed either ∆*phcA‐* or ∆*chpA*‐specific gene expression. These results suggest that ChpA has PhcA‐independent effects on the regulation of these genes.

Deletion of *chpA* significantly reduces the bacterial population in tomato plants, leading to significantly reduced virulence (Corral et al., 2020; Shen et al., 2020), consistent with the results of our virulence assays. However, different from Δ*phcA*, a smaller number of Δ*chpA* cells infected the intercellular spaces of the cortex, leading to infectivity to the xylem vessels as described by Corral et al. (2020) and Shen et al. (2020). The colonization of intercellular spaces of the cortex and xylem vessels by OE1‐1 may thus be affected by QS‐dependent genes but not ChpA‐dependent genes. We previously reported that QS‐deficient mutants lose their ability to infect cell wall‐degraded cortical cells and form mushroom‐shaped biofilms (mBFs) in the cortical cells (Inoue et al., 2023). The transcriptome analyses revealed significantly reduced transcript levels of plant cell wall‐degrading enzyme genes in Δ*chpA* compared with OE1‐1, similar to Δ*phcA*. The microscopic analyses confirmed that Δ*chpA* had lost its ability to degrade cortical cell walls and could not infect cell wall‐denatured cortical cells and form mBFs, which ultimately significantly reduced its virulence. It is thus thought that mBF formation in cell wall‐degraded cortical cells dependent on both PhcA and ChpA is required for virulence of OE1‐1.

The RSSC strains bear type IV pili, which are surface structures associated with a hypothetical pilus‐mediated chemotaxis pathway encoded by *pil*‐*chp* genes (Kang et al., 2002; Siri et al., 2014). The regulator ChpA controls twitching motility in strain GMI1000, suggesting that ChpA is a regulator of type IV pilus assembly (Corral et al., 2020). The dendrogram of swarming‐related *pil* genes based on the transcriptome data showed significantly reduced transcript levels of *pilA* in Δ*chpA* and Δ*phcA*. In contrast, the expression patterns of *pilD*, *pilZ*, and *pilW* differed between Δ*chpA* and Δ*phcA*. Among the positively and negatively ChpA‐dependent genes, 60.7% and 56.5% were positively and negatively QS‐dependent genes, respectively (Table S3). Interestingly, ChpA was found to be involved in the positive regulation of the negatively PhcA‐regulated gene *ripAB*, encoding a type III effector. Thus, ChpA may be involved not only in the regulation of QS‐dependent genes, but also in the regulation of genes including *pilD*, *pilZ*, and *pilW* by the alternative transcription regulator(s).

Swimming motility is repressed when QS is active. The swimming motility of Δ*chpA* and Δ*phcA* on quarter‐strength M63 medium containing 0.25% wt/vol agar was significantly enhanced compared with that of OE1‐1. However, the dendrogram of swimming motility‐related genes based on the transcriptome data showed that the transcript levels of some flagella biogenesis‐related genes such as *fliE*, *flgA*, *fliH*, *fliI*, and *fliK* differed between Δ*phcA* and Δ*chpA*, but were similar in Δ*phcA* and OE1‐1. It is thus thought that these genes may be regulated by alternative transcription regulator(s) rather than PhcA and that ChpA may participate in this regulation. When incubated on modified CPG medium containing 0.3% wt/vol agar, the Δ*chpA* mutant derived from strain GMI1000 exhibits similar swimming motility to that of the parent strain GMI1000 (Corral et al., 2020). However, in our swimming assay using modified CPG medium containing 0.3% wt/vol agar, the swimming motility of Δ*chpA* was higher than that of OE1‐1. Furthermore, *chpA* deletion in strain OE1‐1 led to a loss in virulence. However, *chpA* deletion in strain GMI1000 led to moderately reduced virulence (Corral et al., 2020) or a loss in virulence (Shen et al., 2020). QS is conserved in all RSSC strains and is required for RSSC virulence (Castillo & Agathos, 2019; Hikichi et al., 2017). It is thus thought that the gene regulation by the alternative transcription regulator(s) may affect RSSC virulence independently of QS, and the regulatory effects of ChpA on gene expression may differ among RSSC strains.

In response to iron limitation, genes involved in bacterial siderophore production and uptake are derepressed, leading to the production of siderophores and appropriate uptake proteins (Andrews et al., 2003; Ratledge & Dover, 2000). Compared with OE1‐1, Δ*phcA* exhibited significantly enhanced siderophore‐mediated iron acquisition activity, whereas Δ*chpA* exhibited significantly reduced siderophore‐mediated iron acquisition activity. Though hierarchical clustering with a heatmap representing the global gene expression pattern in OE1‐1, ∆*phcA*, and ∆*chpA* showed that the effect of ChpA on gene expression is close to that of PhcA, the effects are different for some gene sets. Interestingly, cluster II, with genes up‐regulated in ∆*phcA* but not in ∆*chpA*, showed significant enrichment of the term “siderophore (GO:0019290)”. Therefore, ChpA may be involved in the regulation of siderophore‐mediated iron acquisition activity, independently of QS.

Results in the present study suggest that ChpA is involved in the regulation of about 90% of QS‐dependent genes, thereby contributing to the behaviour in host plant roots and the virulence of strain OE1‐1. ChpA is thus involved in the gene regulation required for virulence of OE1‐1, though how ChpA regulates these genes remains unclear. To explore the role of ChpA in gene regulation, in vitro experiments with purified proteins are required. Such analyses will shed further light on the regulation of not only PhcA‐dependent virulence‐related but also alternative transcription regulator(s)‐related genes.

## 4 EXPERIMENTAL PROCEDURES

### 4.1 Bacterial strains, plasmids, and growth conditions

All *R. pseudosolanacearum* strains were routinely grown in quarter‐strength M63 medium at 30°C. *Escherichia coli* strains were grown in lysogeny broth medium (Hanahan, 1983) at 37°C. Gentamycin (50 μg/mL) was used in selective media.

### 4.2 General DNA manipulations

Isolation of genomic DNA, plasmid DNA manipulations, PCR, and Southern blot analyses were performed using standard techniques (Sambrook et al., 1989). OE1‐1 was transformed by electroporation (Kanda et al., 2003). Double‐stranded DNA sequencing templates were prepared with the GenElute Plasmid Miniprep Kit (Sigma). Sequences were determined using an Automated DNA Sequencer (ABI Prism 3100‐Avant Genetic Analyzer, Applied Biosystems). DNA sequence data were analysed using DNASIS‐Mac software (Hitachi Software Engineering).

### 4.3 Generation of the *chpA* ‐deletion mutant

A 671‐bp DNA fragment (delta‐1) was amplified by PCR using the genomic DNA of OE1‐1 (NCBI reference sequence: NZ_CP009764.1) as the template and the primers 0672‐1‐FW (5′‐CGggatccAGATTCAGGACACCTCCAAGCG‐3′) with a BamHI site (lowercase letters) and 0672‐1‐RV (5′‐GCCCGTTCACATGAGGGCTTCCAAAAAGCGTG‐3′). A 704‐bp DNA fragment (delta‐2) was likewise amplified from OE1‐1 using the primers 0672‐2‐FW (5′‐AAGCCCTCATGTGAACGGGCGACGCGC‐3′) and 0672‐2‐RV (5′‐CCCaagcttTGTAGACGCTGGGATGGTGG‐3′) with a HindIII site (lowercase letters). Using the delta‐1 and delta‐2 sequences as templates, a 1355‐bp DNA fragment was PCR‐amplified using the primers 0672‐1‐FW and 0672‐2‐RV and then digested with BamHI (Takara Bio) and HindIII (Takara Bio) to release a 1.4‐kb fragment, which was ligated into the BamHI and HindIII sites of the pK18mobsacB vector (Kvitko & Collmer, 2011) to produce a pdelta‐chpA recombinant plasmid. This plasmid was electroporated into OE1‐1 competent cells, which were prepared as described by Mori et al. (2016). A kanamycin‐sensitive, sucrose‐resistant recombinant, Δ*chpA* (Table 1), was then selected. To verify the actual deletion of *chpA* (RSc0672), a 1823‐bp DNA fragment was PCR‐amplified using the primers 0672‐SQ‐FW (5′‐AGCAGACCGCCGAGCAG‐3′) and 0672‐SQ‐RV (5′‐CACATCTTCGTCTTCCAGCAGCAC‐3′). DNA sequences of this fragment were analysed using 0672‐SQ‐FW and 0672‐SQ‐RV as primers.

### 4.4 Generation of a *chpA* ‐deletion mutant transformed with native *chpA*

The 7496‐bp DNA fragment was amplified by PCR from OE1‐1 using the primers 0672‐1‐FW and 0672‐2‐RV and then digested with BamHI and HindIII to yield a 7.5‐kb fragment. The 7.5‐kb fragment was ligated into the BamHI and HindIII sites of the pUC18‐mini‐Tn*7*T‐Gm vector (Choi et al., 2005) to produce pUC18‐mini‐Tn*7*T‐Gm‐chpA. This plasmid was electroporated into Δ*chpA* competent cells with a Tn*7* transposase expression vector pTNS2 (Choi et al., 2005). Finally, a gentamycin‐resistant transformant, *chpA*‐comp (Table 1), was selected.

### 4.5 Transcriptome analysis based on RNA‐seq

Total RNA was extracted from *R. pseudosolanacearum* strains grown in quarter‐strength M63 until OD_600_ = 0.3 with a High Pure RNA Isolation kit (Roche Diagnostics). Ribosomal RNA was eliminated from the extracted total RNA using a Ribo‐Zero rRNA Removal kit (Gram‐negative bacteria; Illumina) as previously described (Hayashi, Kai, et al., 2019). Then, oriented, paired‐end RNA‐seq (2 × 100 bp) was performed on an Illumina HiSeq 2500 system and DNBSEQ‐G400. The generated reads were trimmed with Cutadapt (Martin, 2011; v. 1.1; http://code.google.com/p/cutadapt/) and Trimmomatic (Bolger et al., 2014; v. 0.32; http://www.usadellab.org/cms/?page=trimmomatic) and then mapped to the GMI1000 genome with the TopHat program (Trapnell et al., 2009; v. 2.0.10; http://tophat.cbcb.umd.edu/). Three independent biological replicates were analysed per strain.

### 4.6 Differential gene expression analysis

Statistical analysis of the RNA‐seq data was performed using R cran (R Core Team, 2022). Genes with zero counts in at least one OE1‐1 sample in the raw count data set were excluded. The RNA‐seq read counts of the remaining genes were normalized using the function calcNormFactors (trimmed mean of M‐values normalization) using the package edgeR (Robinson et al., 2010). To extract genes with significant changes in transcript levels, the following thresholds were applied: *q* < 0.05 and log_2_(fold change) ≥ |2|. The false discovery rate (*q* value) was calculated from *p* values estimated by edgeR using the Benjamini–Hochberg method (Benjamini & Hochberg, 1995). For PCA, the R built‐in function prcomp was used with the “scale = TRUE” option. Hierarchical clustering of all normalized mean transcript values based on their relative transcript levels (counts per million) was performed using Cluster v. 3.0 software (de Hoon et al., 2004) or the R package hclust. The average of three replicates per strain was calculated. Heatmaps were created with TreeView (Eisen et al., 1998) or the R package pheatmap. GO terms were obtained from the QuickGO database (https://www.ebi.ac.uk/QuickGO/annotations) with the *R. solanacearum* GMI1000 gene annotation data. GO enrichment analysis was performed using the R package goseq (Young et al., 2010).

### 4.7 RT‐qPCR analyses

Total RNA was extracted from *R. pseudosolanacearum* strains grown in quarter‐strength M63 until OD_600_ = 0.3 with the High Pure RNA Isolation kit. An RT‐qPCR assay with gene‐specific primers (Table S6) was carried out using the SYBR GreenER qPCR reagent system (Invitrogen) using a 7300 Real‐Time PCR system (Applied Biosystems) as previously described (Hayashi, Kai, et al., 2019). All values were normalized against the transcript level of *rpoD*, which was used as an internal standard for each cDNA sample. The *rpoD* transcript level did not differ significantly among the *R. pseudosolanacearum* strains. The experiments were performed with three biological replicates and two technical replicates. Mean values were compared and significant differences between *R. pseudosolanacearum* strains were detected by Student's *t* test in Microsoft Excel.

### 4.8 QS‐dependent phenotypes

The in vitro biofilm formation of *R. pseudosolanacearum* strains grown without shaking in quarter‐strength M63 was analysed as previously described (Mori et al., 2016). The biofilm was quantified on the basis of absorbance at 550 nm (A_550_). The resulting value was normalized to the number of cells (OD_600_). Three replicate experiments were conducted using independent samples, with seven technical replicates per experiment. Means were analysed for significant differences between *R. pseudosolanacearum* strains by analysis of variance followed by Tukey–Kramer's honestly significant difference (HSD) test (*p* < 0.05).

The production of EPS I by *R. pseudosolanacearum* cells grown on quarter‐strength M63 solidified with 1.5% agar was quantitatively analysed by ELISA (Agdia) as previously described (Mori et al., 2016). EPS I production was quantified by measuring A_650_. Three replicate experiments were conducted using independent samples, with seven technical replicates per experiment. Means were analysed for significant differences between *R. pseudosolanacearum* strains by analysis of variance followed by Tukey–Kramer'sHSD test (*p* < 0.05).

For the swimming motility assay, overnight cultures of *R*. *pseudosolanacearum* strains were washed with distilled water and then diluted to a cell density of 5 × 10^5^ cfu/mL, and 5‐μL aliquots of cell suspensions were added to the centre of quarter‐strength M63 medium solidified with 0.25% wt/vol agar. The swimming‐area diameters of *R. pseudosolanacearum* strains were measured at 48 h postinoculation (Mori et al., 2018). Three replicate experiments were conducted using independent samples, with five technical replicates per experiment. Means were analysed for significant differences between *R. pseudosolanacearum* strains by analysis of variance followed by Tukey–Kramer's HSD test (*p* < 0.05).

To determine the effect of incubation on modified CPG medium with 0.3% wt/vol agar, 5‐μL aliquots of cell suspensions were added to the centre of modified CPG medium solidified with 0.3% wt/vol agar as described by Corral et al. (2020). The swimming‐area diameters of *R. pseudosolanacearum* strains were measured at 48 h postinoculation. The experiment was repeated three times, each with five technical replicates. Means were analysed for significant differences between *R. pseudosolanacearum* strains by analysis of variance followed by Tukey–Kramer's HSD test (*p* < 0.05).

To investigate the in vitro growth rate, overnight cultures of *R. pseudosolanacearum* strains were diluted to OD_600_ = 0.01 with quarter‐strength M63 and grown with shaking using a compact rocking incubator (TVS062CA, ADVANTEC). The in vitro growth rate of bacterial strains was quantified based on OD_600_ of the bacterial culture. Three replicate experiments were conducted using independent samples, with three technical replicates per experiment. Means were analysed for significant differences between *R. pseudosolanacearum* strains by analysis of variance followed by Tukey–Kramer's HSD test (*p* < 0.05).

### 4.9 Siderophore‐mediated iron acquisition activity

The siderophore‐mediated iron acquisition activity of *R. pseudosolanacearum* strains was analysed as previously described (Takemura et al., 2021). In brief, *R. pseudosolanacearum* strains were incubated in PY medium (5 g/L polypeptone, 2 g/L yeast extract) for 18 h at 30°C and adjusted to a concentration of 2 × 10^9^ cfu/mL with 0.1 M PIPES buffer (pH 6.5). After 6 h of incubation, each culture was filtered through a 0.2‐μm pore filter. Next, a 100‐μL aliquot of a culture, or PIPES buffer alone as a reference, was added to 100 μL chromazurol S (CAS) solution (2.4 mM hexadecyl‐trimethyl ammonium bromide, 0.06 mM FeCl_3_, 0.6 mM HCl, 0.6 mM CAS in PIPES buffer). Absorbance at 630 nm (A_630_) was measured after incubation for 30 min at 30°C. Siderophore activity was calculated by subtracting the absorbance value of the reference from the total absorbance. Three replicate experiments were conducted using independent samples, with eight technical replicates per assay. Means were analysed for significant differences between *R. pseudosolanacearum* strains by analysis of variance followed by Tukey–Kramer's HSD test (*p* < 0.05).

### 4.10 Virulence assays

Eight‐week‐old tomato plants (*Solanum lycopersicum* ‘Ohgata‐Fukuju’) were inoculated with *R. pseudosolanacearum* strains (10^8^ cfu/mL) by a root‐dip method as previously described (Hayashi, Kai, et al., 2019). Plants were monitored daily for wilting symptoms, which were rated according to the following disease index scale: 0, no wilting; 1, 1%–25% wilting; 2, 26%–50% wilting; 3, 51%–75% wilting; 4, 76%–99% wilting; 5, dead. For each bacterial strain, three independent groups were tested, with 12 technical replicates per group. Means were analysed for significant differences between *R. pseudosolanacearum* strains by analysis of variance followed by Tukey–Kramer's HSD test (*p* < 0.05).

The infection process of *R. pseudosolanacearum* strains in tomato roots was observed using the tomato–*R. solanacearum* model system (Inoue et al., 2023). In brief, seeds of tomato (*S. lycopersicum* ‘Ohgata‐Fukuju’) plants were sterilized with 70% ethanol solution for 20 s and with 1% sodium hypochlorite solution for 20 min. The seeds were sown on 1% agar in Petri dishes at 30°C under dark and axenic conditions. Four days after sowing, seedlings with approximately 20‐mm long roots were placed on agar plates, and 200 μL of OE1‐1 suspension at 10^8^ cfu/mL was added to the centre of the plate, 10 mm away from the tomato roots. The tomato seedlings were then incubated at 30°C under dark and axenic conditions. Root pieces were excised 4 mm from the tip, fixed in half‐strength Karnovsky's fixative overnight at 4°C, and then post‐fixed in 1% osmium tetroxide for 1 h at room temperature (Karnovsky, 1965). The specimens were dehydrated in an ethanol series (three times at 30%, 50%, 70%, 80%, 90%, and 100%) for 10 min each at 25°C and then embedded in Quetol‐812 resin (Nisshin EM). Serial semithin sections (800 nm thick) were cut with a diamond knife, mounted on glass slides, stained with toluidine blue, and observed under a BX53 optical microscope equipped with a DP74 camera (Olympus).

To assay the influence of the *chpA* deletion on the infectivity of *R. pseudosolanacearum* strains in cortical cells, we counted the number of cortical cells with a nucleus and infected with *R. pseudosolanacearum* adjacent to the epidermis in the elongation zone using microscopic imaging data of serial semithin sections of tomato roots after 48 HOI with *R. pseudosolanacearum*. Means were analysed for significant differences between *R. pseudosolanacearum* strains by analysis of variance followed by Tukey–Kramer's HSD test (*p* < 0.05).

## CONFLICT OF INTEREST STATEMENT

The authors declare that they have no conflicts of interest.

## Supporting information


**FIGURE S1.** Genomic location of *chpA* in the *Ralstonia pseudosolanacearum* strain OE1‐1 genome and depiction of plasmid construction for *chpA* knockout.

**FIGURE S2.** Global effect of ChpA on gene expression in *Ralstonia pseudosolanacearum*. A principal component analysis plot for the transcriptome data of negatively quorum sensing‐dependent genes in OE1‐1 and the *phcA*‐deletion (Δ*phcA*), *phcR*‐deletion (Δ*phcR*), *phcQ*‐deletion (Δ*phcQ*), and *chpA*‐deletion (Δ*chpA*) mutants.

**TABLE S1.** RNA‐sequencing data for transcripts of genes in *Ralstonia pseudosolanacearum* strain OE1‐1 and the *phcB*‐deletion (Δ*phcB*), *phcA*‐deletion (Δ*phcA*), and *chpA*‐deletion (Δ*chpA*) mutants grown in quarter‐strength M63 medium, and predicted functions of putative gene products.

**TABLE S2.** RNA‐sequencing data for transcripts of quorum sensing‐dependent genes in *Ralstonia pseudosolanacearum* strain OE1‐1 and the *phcB*‐deletion (Δ*phcB*), *phcA*‐deletion (Δ*phcA*), and *chpA*‐deletion (Δ*chpA*) mutants grown in quarter‐strength M63 medium, and predicted functions of putative gene products.

**TABLE S3.** RNA‐sequencing data for transcripts of ChpA‐regulated genes in *Ralstonia pseudosolanacearum* strain OE1‐1 and the *phcB*‐deletion (Δ*phcB*), *phcA*‐deletion (Δ*phcA*), and *chpA*‐deletion (Δ*chpA*) mutants grown in quarter‐strength M63 medium, and predicted functions of putative gene products.

**TABLE S4.** RNA‐sequencing data for transcripts of genes involved in twitching motility governed by type IV pili in *Ralstonia pseudosolanacearum* strain OE1‐1 and the *phcB*‐deletion (Δ*phcB*), *phcA*‐deletion (Δ*phcA*), and *chpA*‐deletion (Δ*chpA*) mutants grown in quarter‐strength M63 medium, and predicted functions of putative gene products.

**Table S5.** RNA‐sequencing data for transcripts of swimming motility‐related genes in *Ralstonia pseudosolanacearum* strain OE1‐1 and the *phcB*‐deletion (Δ*phcB*), *phcA*‐deletion (Δ*phcA*), and *chpA*‐deletion (Δ*chpA*) mutants grown in quarter‐strength M63 medium, and predicted functions of putative gene products.

**TABLE S6.** Primers used in quantitative real‐time polymerase chain reaction assays.

## Data Availability

The data that support the findings of this study are available from the corresponding author upon reasonable request. RNA‐seq data are available in the NCBI SRA repository at https://www.ncbi.nlm.nih.gov/sra/ under accession codes DRR438005, DRR438006, and DRR438007 (WT: OE1‐1); DRR438010, DRR450853, and DRR450854 (∆*phcA*); DRR450855, DRR450856, and DRR450857 (∆*phcQ*); DRR450858, DRR450859, and DRR450860 (∆*phcR*); and DRR450861, DRR450862, and DRR450863 (∆*chpA*).
