# Supplementary figures and images for: The transcription regulator ChpA affects the global transcriptome including quorum sensing‐dependent genes in Ralstonia pseudosolanacearum strain OE1‐1
Source: Mol Plant Pathol. 2023 Jul 14;24(11):1370–84. doi: 10.1111/mpp.13374 (PMC10576176; doi:10.1111/mpp.13374)

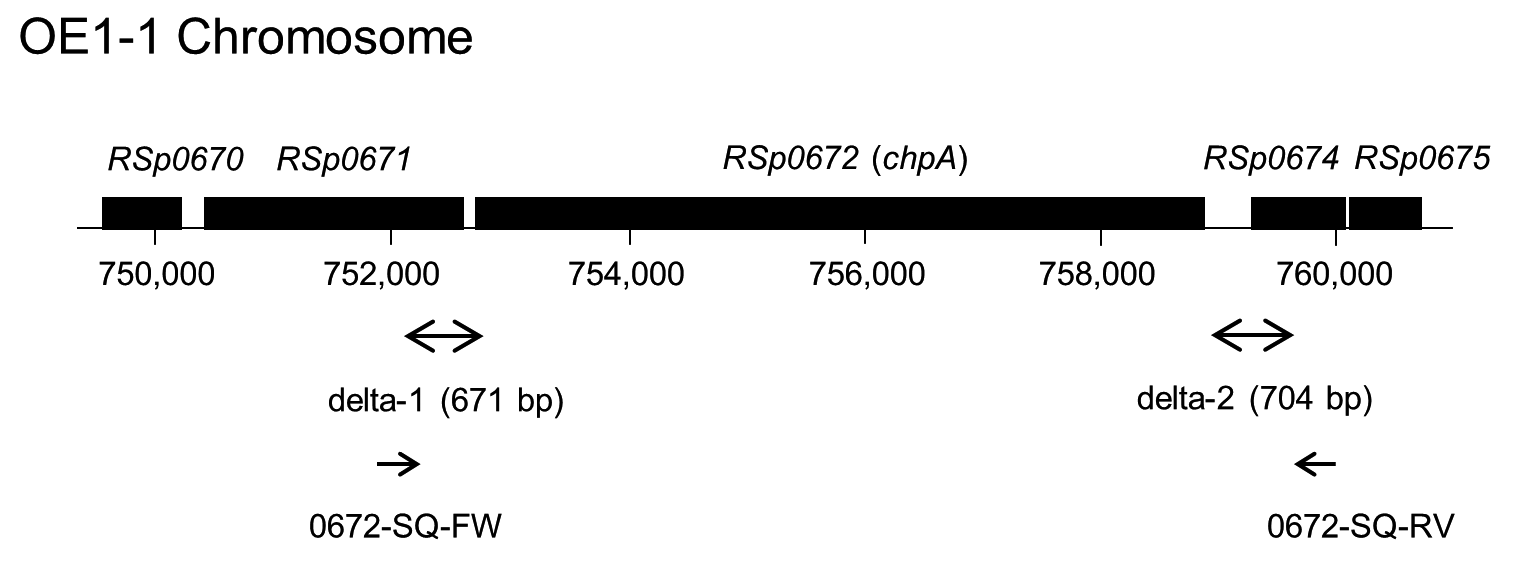

Supplement: Supplementary file 1 — FIGURE S1. Genomic location of chpA in the Ralstonia pseudosolanacearum strain OE1‐1 genome and depiction of plasmid construction for chpA knockout. [file MPP-24-1370-s008.tif]

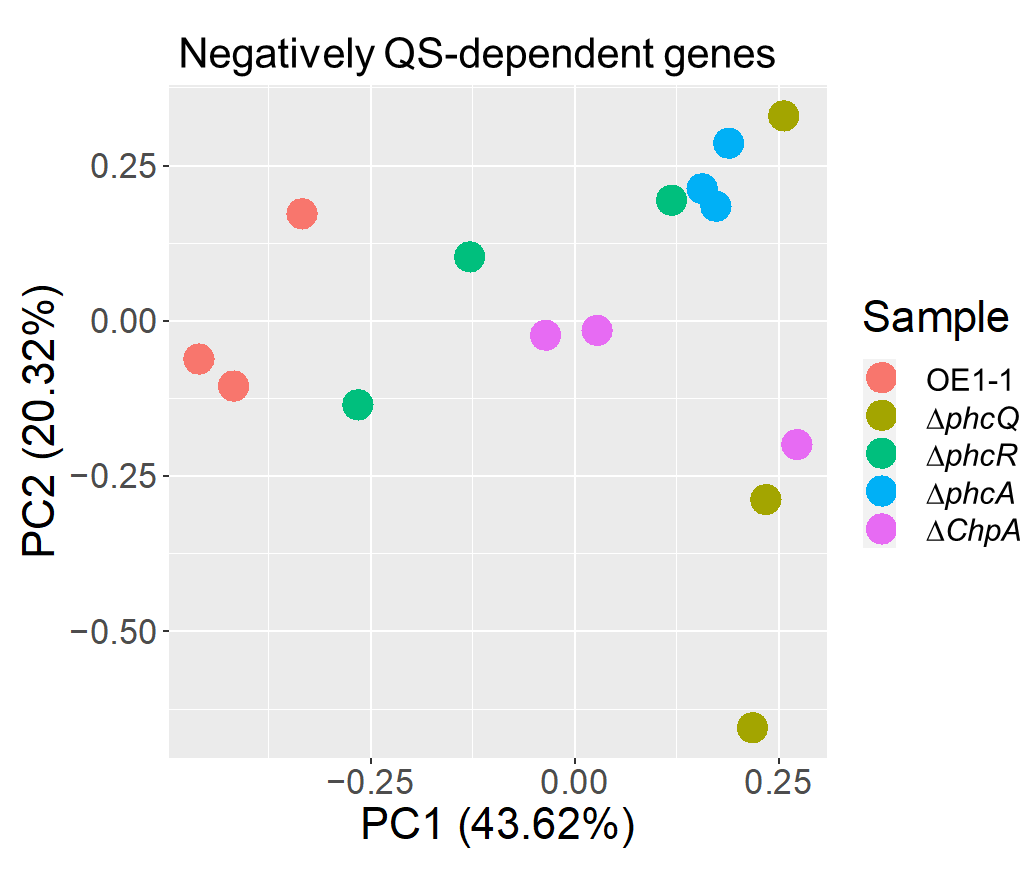

Supplement: Supplementary file 2 — FIGURE S2. Global effect of ChpA on gene expression in Ralstonia pseudosolanacearum. A principal component analysis plot for the transcriptome data of negatively quorum sensing‐dependent genes in OE1‐1 and the phcA‐deletion (ΔphcA), phcR‐deletion (ΔphcR), phcQ‐deletion (ΔphcQ), and chpA‐deletion (ΔchpA) mutants. [file MPP-24-1370-s006.tiff]
